# Magnetic Hydrogel Composite for Wastewater Treatment

**DOI:** 10.3390/polym14235074

**Published:** 2022-11-22

**Authors:** Bidita Salahuddin, Shazed Aziz, Shuai Gao, Md. Shahriar A. Hossain, Motasim Billah, Zhonghua Zhu, Nasim Amiralian

**Affiliations:** 1 Australian Institute for Bioengineering and Nanotechnology (AIBN), The University of Queensland, Brisbane, QLD 4072, Australia; 2 School of Chemical Engineering, The University of Queensland, Brisbane, QLD 4072, Australia; 3 School of Mechanical and Mining Engineering, The University of Queensland, Brisbane, QLD 4072, Australia

**Keywords:** functional hybrid composite, hydrogel composite, nanocellulose, magnetic nanoparticle, wastewater treatment

## Abstract

Nanocomposite hydrogels are highly porous colloidal structures with a high adsorption capacity, making them promising materials for wastewater treatment. In particular, magnetic nanoparticle (MNP) incorporated hydrogels are an excellent adsorbent for aquatic pollutants. An added advantage is that, with the application of an external magnetic field, magnetic hydrogels can be collected back from the wastewater system. However, magnetic hydrogels are quite brittle and structurally unstable under compact conditions such as in fixed-bed adsorption columns. To address this issue, this study demonstrates a unique hydrogel composite bead structure, providing a good adsorption capacity and superior compressive stress tolerance due to the presence of hollow cores within the beads. The gel beads contain alginate polymer as the matrix and MNP-decorated cellulose nanofibres (CNF) as the reinforcing agent. The MNPs within the gel provide active adsorption functionality, while CNF provide a good stress transfer phenomenon when the beads are under compressive stress. Their adsorption performance is evaluated in a red mud solution for pollutant adsorption. Composite gel beads have shown high performance in adsorbing metal (aluminium, potassium, selenium, sodium, and vanadium) and non-metal (sulphur) contaminations. This novel hybrid hydrogel could be a promising alternative to the conventionally used toxic adsorbent, providing environmentally friendly operational benefits.

## 1. Introduction

Global water pollution management involves decisive and innovative materials and technologies that can successfully remove pollutants from wastewater [1,2,3]. The existing technologies for removing pollutants from wastewater are helpful but far from perfect regarding efficiency, their environmental footprint, and economic feasibility. The standard techniques to treat wastewater are adsorption, chemical precipitation, ion exchange, membrane separation, coagulation–flocculation, and electrochemical separation [4,5,6,7,8,9]. These techniques have noteworthy shortcomings and constraints, comprising the partial elimination of pollutants, expensive operation and high energy consumption, and production of toxic chemical sludge [10]. Although adsorption has some limitations, such as the high cost of adsorbent, difficulty separating the adsorbent from dye, and low surface area [11], it is still the most extensively used technique in wastewater treatment. An efficient adsorbent eliminates most contaminants, while it can be regenerated easily using solvents or electrochemical treatments [12,13,14]. However, current commercial adsorbents such as activated carbon, biochar, zeolites, and kaolin are rigid and required in bulk for effective pollutant adsorption [15,16,17,18]. Commercial adsorbents also pose a high risk of water contamination by themselves.

Regarding the adsorbent materials, functional composites with collective physicochemical properties could lead to an effective adsorption solution. In recent years, nanocomposite hydrogels have been broadly utilised in many adsorption-based water purification applications [19,20,21] where a polymer is usually used as the continuous matrix phase and nanoscale materials are used as the filler. Hydrogels have three-dimensional (3D) porous networks of hydrophilic polymer chains that can absorb and retain a large volume of water [22,23]. Utilising biopolymer-derived biocompatible hydrogels such as alginate, cellulose, and chitosan offers an environmentally friendly and safe process for pollution remediation [24]. Incorporating nanoparticles into hydrogel results in enhanced adsorption efficiency due to their high surface area, as well as reduced operational costs due to their higher efficiency. For instance, the application of magnetic nanoparticles (MNP) in wastewater treatment showed promising outcomes about fifteen years ago [25]. MNPs exhibit a high surface-to-volume ratio to capture a higher content of contaminant. MNPs can be loaded into contaminants and separated easily by applying an external magnetic field. Moreover, they can provide varying functionalities when chemically modified, such as coating with inorganic shells and/or treating with organic molecules, providing an increased affinity for capturing contaminants [26,27]. Magnetic nanoparticles such as iron oxide can also be regenerated through acid treatment, thus decreasing the economic burden [28,29]. Over the years, wide-ranging toxicity studies (in vitro/in vivo) and a huge database have been developed regarding the toxicity of magnetic particles [30,31] that support improving the methods for wastewater treatment without having any environmentally harmful wastes or by-products.

Although MNP-incorporated hydrogels are incredibly beneficial for adsorption applications, these composite materials are significantly brittle and structurally unstable under compression stress [32,33]. For example, iron oxide incorporated alginate hydrogel beads have been promising for sulphur removal from wastewater, but the extent of nanoparticle monodispersity is relatively low, causing uneven mechanical properties and structural failure under stress. Such low mechanical properties are not expected when the composite materials are used in a packed column purifier [34,35]. One way to overcome the low mechanical properties is to use a carrier for the MNPs before incorporating them with the polymer matrix. Because nanoscale fibres have a low active adsorption volume, they can be considered potential carriers of surface MNPs [36]. Natural nanofibres such as cellulose nanofibres (CNF) can be used as a template to synthesise nanoparticles with uniform size and distribution [37]. CNFs generally have diameters ranging 4–20 nm and lengths of up to several microns, exhibiting very high aspect ratios, which is critical to improving the toughness of the hydrogel system [38]. The nanofibres decorated with surface MNPs can then be used as a hydrogel matrix reinforcing agent while being biocompatible and cost-effective.

In this paper, sustainable nanocomposite hydrogel beads are demonstrated that comprise CNFs decorated with MNPs as the filler and alginate as a hydrogel matrix. Bleached sugarcane pulp is used to extract the CNF, and the synthesis of iron oxide (Fe_3_O_4_) MNPs was carried out on the surface of the nanofibres through the co-precipitation process. The physical, mechanical, and microstructural properties of the composite beads are investigated, and their adsorption performance is evaluated in aqueous media for pollutant removal.

## 2. Materials and Methods

### 2.1. Materials

The raw sugarcane mulch was received from Sugar Research Australia Ltd. (Brisbane, Australia). Materials used in this study include chemicals for pulping sugarcane: sodium hydroxide (Chem-supply, Adelaide, Australia), glacial acetic acid (Merck, Rahway, NJ, USA), and sodium chlorite-Technical Grade 80% (Sigma-Aldrich, St. Louis, MO, USA); chemicals for the in situ synthesis of iron oxide: ammonium hydroxide 25% (*w*/*w*) (Sigma-Aldrich), ferric (III) chloride hexahydrate 96% (Sigma-Aldrich), and ferrous (II) chloride 98% (Sigma-Aldrich); and materials for the gel composite matrix: sodium alginate (Sigma-Aldrich). Deionised (DI) water was used in all experimental works.

### 2.2. Fabrication of Hybrid Hydrogel Composites

**Figure 1:**
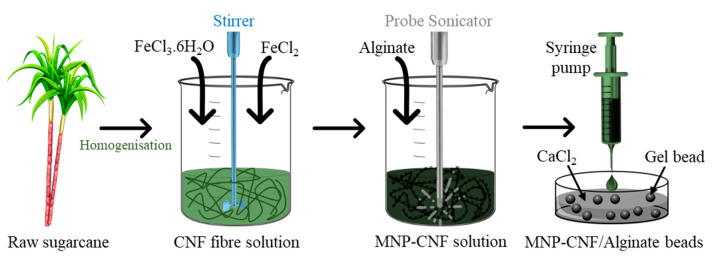
Schematic illustration of the fabrication method of magnetic nanocellulose incorporated alginate (MNP–CNF/alginate) beads.

Hybrid hydrogel beads were prepared in a three-step process: (1) preparation of the CNF, (2) in situ precipitation of the MNPs on the CNF, and (3) solution mixing of the CNF and MNP–CNF with alginate for preparing CNF/alginate and MNP–CNF/alginate gel beads separately. Figure 1 shows the schematic illustration of the fabrication method of the gel beads.

For preparing the cellulose nanofibres, bleached sugarcane pulp was produced using the method reported before [37,39]. Washed and ground sugarcane fibres were treated with a 2% (*w*/*v*) sodium hydroxide solution using a 10:1 solvent to sugarcane ratio at 80 °C for two hours, followed by rinsing with 60 °C water. Alkali-treated fibres were then bleached twice using an acidic solution of 1% (*w*/*v*) sodium chlorite (pH = 4, the pH decreased with glacial acetic acid) at 70 °C for one hour at a 30:1 solvent to fibres ratio. CNF were prepared by passing a 0.7% (*w*/*v*) dispersion of bleached sugarcane pulp through a high-pressure homogeniser (GEA Niro-Soavi Panda NS1001 L 2 K Homogeniser, Brisbane, Australia) for one pass at 800 bar and three passes at 1100 bar.

Iron oxide (Fe_3_O_4_) MNPs were synthesised on the surface of the CNF using the co-precipitation of two iron salts: ferric (III) chloride hexahydrate and ferrous (II) chloride. A similar method was also demonstrated in our previous publication on magnetic nanocellulose [37]. For 30 min, 0.84% (*w*/*v*) CNF was first dispersed in deionised water under purging with nitrogen gas. Then, 2.2 g FeCl_3_·6H_2_O was added to the nanofibre dispersion, and the mixture was stirred for one hour, followed by adding 0.45 g FeCl_2_ to the mixture and stirring for another hour under the nitrogen flow. Subsequently, 10 mL of the 25% NH_4_OH solution was added dropwise into the dispersion and stirred for an hour until the Fe_3_O_4_ particles were formed and the mixture’s colour turned black. The magnetic cellulose nanofibres, MNP–CNF, were washed thrice with ethanol and water using a centrifuge at 20,000 rpm for 15 min. The concentration of CNF was 0.7% (*w*/*v*), while the molar ratio of [Fe^3+^]/[Fe^2+^] was kept at 2. All experiments were performed at room temperature. The average size of our MNPs is in the range of 9- 14 nm with a narrow distribution. The presence of CNF during the synthesis of MNPS facilitates the formation of small nanoparticles with a narrow size distribution, possibly by evenly distributing the metal ions precipitated into each nanoparticle.

For preparing the nanocellulose and magnetic nanocellulose incorporated alginate hydrogels (CNF/alginate and MNP–CNF/alginate), an aqueous alginate solution of (1.9% *w*/*w*) was initially mixed with 0.84% (*w*/*v*) CNF and 2.15% (*w*/*v*) MNP–CNF solution separately at 50:50. An ultrasonic probe (Sonics VC 505, Newtown, CT, USA) for 20 min (Pulse: 40 s ON, 20 s OFF) was used at 40% amplitude while keeping the solution in an ice bath to prevent an unwanted temperature rise. Afterwards, composites were dripped with a syringe pump at a 1 mL/min flowrate (KDS 100, KD Scientific Inc, Holliston, MA, USA) into an aqueous 1.1% (*w*/*w*) CaCl_2_ solution for around 15 min to form spherical gel beads [40,41]. The beads were then filtered out from the solution and washed with DI water to remove excess/unreacted Ca^2+^ from the surface. The composite beads were dried in a convection oven at 60 (±0.5) °C for 24 h. A 1.27 mm dispenser needle was used that allowed the gel droplet to expand spherically at the exit to produce a hollow core within the sphere.

### 2.3. Materials Characterisation

#### 2.3.1. Morphological Characterisation

The surface and internal structural morphology of the composite gel beads were investigated both using dry- and wet-state microscopy. A bright-field optical microscope (Olympus BX51, Tokyo, Japan) was used to study the opacity of the dry beads that provides basic information on the qualitative extent of the monodispersity of the nanomaterials within the gel. Dark-field microscopy was used to evaluate the bead size before and after swelling tests. A scanning electron microscope (SEM, Hitachi SU3500, Ibaraki, Japan) was used (accelerating voltage, 10 KV) to understand the nanomaterials’ interaction with the alginate matrix, which is crucial to have a better filler–matrix stress transfer capability. Before SEM observations, samples were sputter-coated with platinum (~15 nm coating thickness). Section-cut beads were also microscopically observed to confirm the presence of a hollow core within the beads. A highly porous matrix structure is desirable for better volumetric transport of the adsorbates that are magnetically attracted by the MNPs.

#### 2.3.2. Swelling Characterisation

The swelling ratio of the gel beads was determined at 22 °C by measuring the weight change. The beads were immersed in water for 60 h at 22 °C, and the swelling ratio Q was calculated using:

(1) $$Q=\frac{\left(W_{s}-W_{D}\right)}{W_{D}}\times 100\%$$

$W_{D}$ is the weight of the dry hydrogel, and $W_{s}$ is the weight of the swollen hydrogel.

#### 2.3.3. Thermal and Mechanical Characterisation

Thermogravimetric analysis (TGA) was performed on a thermal analyser (PerkinElmer STA 6000, Waltham, MA, USA) having a range 40–800 °C at a heating rate of 10 °C min^−1^ under the flow of air. The mechanical characterisation of the hydrogels was conducted under compression. A universal mechanical tester (Instron 5584, Norwood, MA, USA) and an advanced video extensometer were used to perform uniaxial compression testing equipped with 100 N load cell and compression plates. The hydrogel samples were placed within the compression plates and compressed with a crosshead speed of 1 mm/min until ~82% compression strain was reached, and then the compression force was gradually released.

### 2.4. Adsorption Characterisation

#### 2.4.1. Pollutant Adsorption from Wastewater

The adsorption of pollutants in the wastewater was carried out in the shaking water bath (100 rpm) at room temperature. About 1 mg of the gel beads was poured into 15 mL wastewater and shaken for 24 h. After that, the filtrate was separated from the gel beads with a 0.45 µm syringe filter. Finally, the metal concentrations in the wastewater before and after adsorption were characterised using ICP-AES by ALS Environmental Division, Brisbane, Australia.

#### 2.4.2. N_2_ and CO_2_ Adsorption

Sorption isotherms of N_2_ at −196 °C and CO_2_ at 0 °C were measured with a TriStar II 3020 apparatus (Micromeritics, Norcross, GA, USA). The samples were degassed at 150 °C and a pressure of 10^−5^ torr for 72 h before the adsorption tests. The N_2_ isotherms were used to determine the Brunauer-Emmett-Teller (BET) specific surface area at a relative pressure in the range of P/P_0_ = (0.05–0.35), with the total pore (<66.4455 nm) volumes at P/P_0_ = 0.97. The pore size distributions were also calculated from the N_2_ and CO_2_ isotherms using the non-local density functional theory (NLDFT) model. The equivalent surface area, limiting micropore volume, and limiting micropore capacity were calculated from the CO_2_ isotherm with the DA method [42].

## 3. Results and Discussion

### 3.1. Material Properties

#### 3.1.1. Morphology

**Figure 2:**
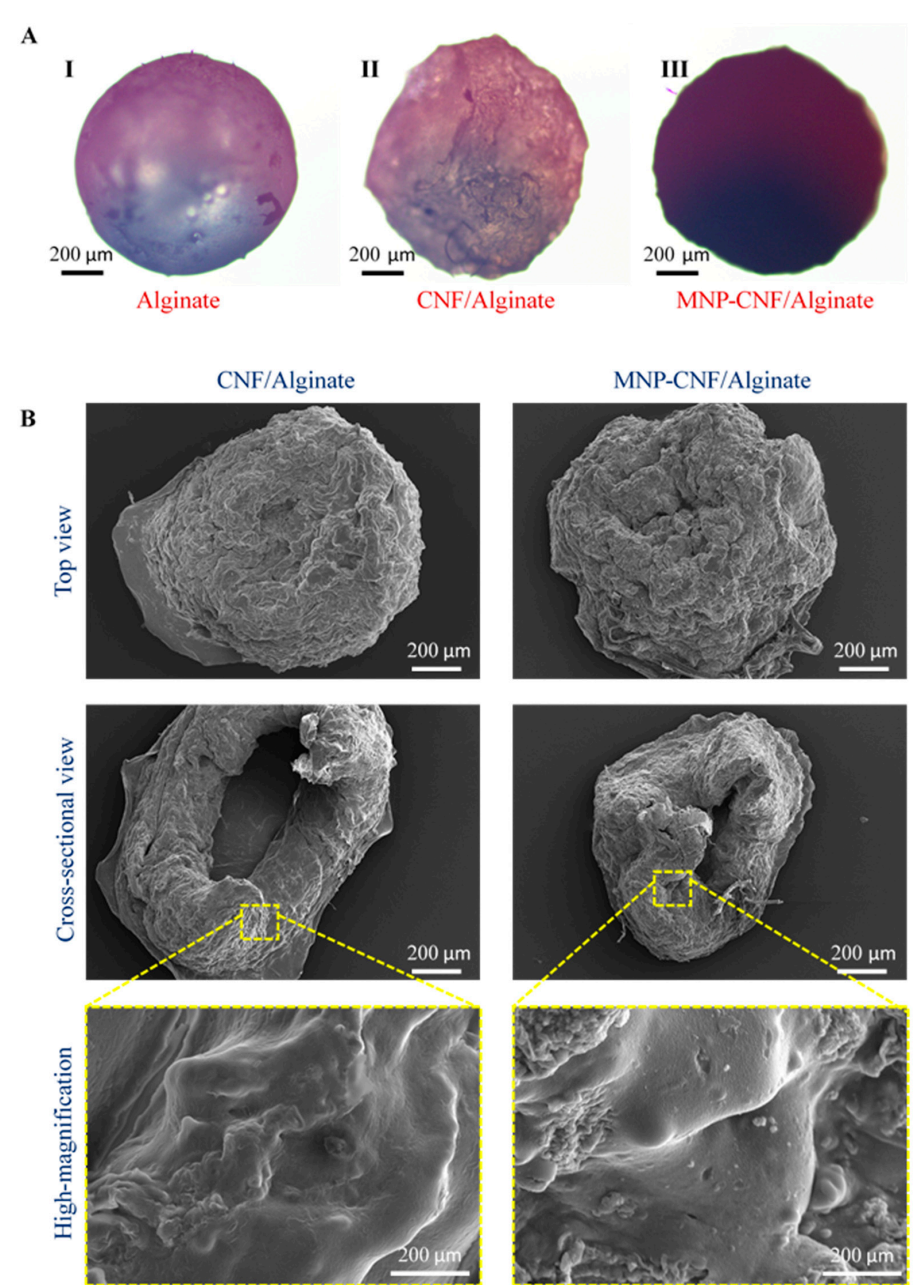
Morphology of dry composite beads: (**A**) Bright-field optical microscopy revealing the opacity and texture of dry beads made of: **I**. alginate only, **II**. nanocellulose incorporated alginate (CNF/alginate) and **III**. magnetic nanocellulose incorporated alginate (MNP–CNF/alginate). (**B**) SEM images of dry beads.

Both dry and swollen composite gel beads were microscopically investigated to study the surface and internal morphology. The external structure and colour changes from the dry alginate to the MNP–CNF/alginate beads as observed with an optical microscope are shown in Figure 2A. The CNF/alginate and MNP–CNF/alginate beads have a uniform shape and size distribution with an average diameter of 3.5 mm and 4 mm, respectively, which was controlled by fixing the flow rate of the nanocomposite solutions through the syringe pump and the distance between the syringe tip and the CaCl_2_ solution. The surface morphology of both nanocomposite beads appeared to be wrinkled and rougher than the pure alginate beads due to the gradual water loss adsorbed by CNF. The cross-sectional images display the internal structure of each bead. Both nanocomposite beads demonstrate a hollow core within their structure. This is attributed to the fast crosslinking of the surface of the alginate droplets in the CaCl_2_ solution when dispensed from the syringe pump. The hollow structure of the beads could improve the loading efficiency of the wastewater, thereby increasing the absorbance properties by trapping more wastewater contamination in the holes. In addition, the hollow structure provides a much larger surface area, allowing a higher surface reactivity and mass transfer, which also is beneficial for contamination adsorption [43]. The SEM images in Figure 2B show a networked structure between the alginate and the nanofibres with no obvious large pore size. The high compressive robustness of these beads could enable their continuous operation when wastewater treatment is conducted through a packed column-based purifier.

#### 3.1.2. Swelling Behaviour

**Figure 3:**
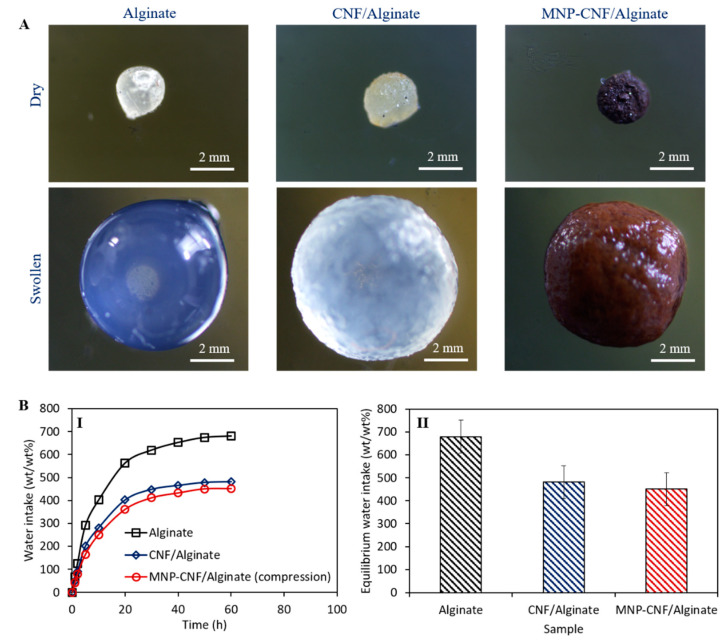
Swelling behaviour of composite gel beads. (**A**) Volume expansion of gel beads while fully swollen. (**B**) Water intake capacity of the gel beads: **I**. Time-dependent water intake curves and **II**. Equilibrium water intake after 60 h of swelling tests.

Figure 3 shows the swelling behaviour of the hybrid gel beads. The microscopic images (Figure 3A) of alginate, CNF/alginate, and MNP–CNF/alginate gel beads illustrate the volume expansion of the beads from the dry to the wet state, providing 300%, 310%, and 280% volumetric expansion, respectively. After immersing the dry beads in deionised water, the bead swelling and the expansion of internal voids enhance the beads’ ability for water adsorption. Hybrid hydrogel bead structures with voids augment the water transport efficiency of gel beads. The maximum water intake capacity was observed for the alginate beads (Figure 3B) due to the highly connected void portion inside the alginate matrix [44]. While comparing the water intake capacity of CNF/alginate with MNP–CNF/alginate, a higher water adsorption capacity was obtained for the CNF/alginate gel beads. This is probably attributable to the fact that the hydroxyl groups on the surface of the nanofibres were partially used as nucleation sites for MNP formation. The iron oxide nanoparticles have a lower hydrophilicity than nanocellulose [37]. Therefore, the lowest water intake was achieved for the MNP–CNF/alginate beads. To confirm the validity of the swelling results, three samples of each bead type were tested and the standard deviations are provided in Figure 3(BII).

#### 3.1.3. Thermal and Mechanical Characterisation

**Figure 4:**
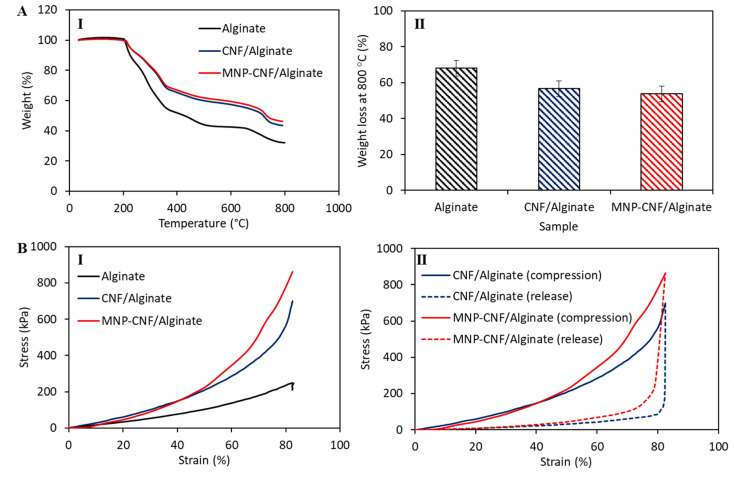
Thermal and mechanical properties of composite gel beads. (**A**) Thermogravimetric test results: (**I**) Thermal degradation of gel beads over 40–800 °C, (**II**) Overall weight loss at 800 °C. (**B**) Compression test results: (**I**) Stress-strain curves of different gel beads showing the differences in stiffness, (**II**). Shape recovery of hybrid gel beads after compression.

The thermal stability of the alginate and nanocomposite beads was evaluated using a TGA tool. Figure 4A shows the multistage thermal degradation for all gel beads with an insignificant difference in the onset temperature of ~210 °C. The highest weight loss was found to be 67% at 800 °C for the alginate beads, whereas the lowest weight loss of 52% was obtained for the MNP–CNF/alginate gel beads. Different weight loss curves indicate the presence of incorporating CNF and Fe_3_O_4_ magnetic nanoparticles into the alginate and CNF/alginate matrix. This incorporation influenced the thermal decomposition of the alginate and CNF/alginate because of the higher thermal stability of CNF due to their higher crystallinity than alginate, the higher thermal stability of iron oxide nanoparticles, and the uniform heat distribution of iron oxide across the CNF/alginate matrix [45,46].

The stress-strain properties of the three different gel beads in the swollen conditions are illustrated in Figure 4B. The pure alginate bead breaks at ~82% compression strain, only sustaining ~247 kPa stress, while the MNP–CNF/alginate gel bead shows the maximum compressive strength of ~853 kPa when compressed deformed by ~82%, which is ~3.5 times higher than the pure alginate bead. This compressive strength is largely higher than previously demonstrated CNF-reinforced hydrogel composites, as shown in Table 1.

The high-stress tolerance is attributed to the reinforcing effect of the cellulose nanofibres that absorb the compressive stress applied to the gel beads; therefore, it is unlikely that a certain extent of compressive stress will form cracks on the bead shell. It is also possible that the incorporation of MNPs resulted in augmented interfacial binding between the gel matrix and CNF, causing increased toughness and excellent energy dissipation capability during compressive loading (Figure 4(BI)) [25]. The presence of MNPs in between the CNF and the gel matrix creates interfacial networks which can bridge gel-rich areas, enhancing the interfacial properties of the nanocomposite beads. In particular, the enhanced interfacial shear strength (IFSS) of the gel beads provides increased toughness during compressive loading [52,53]. As observed (Figure 4(BII)), the stress-strain curve was fully reversible for the CNF/alginate and MNP–CNF/alginate gel beads upon loading (compression) and unloading (release). Three samples were tested, and the loading/unloading process was repeated five times for each sample, implying the reusability of the gel beads.

#### 3.1.4. Porosity

**Figure 5:**
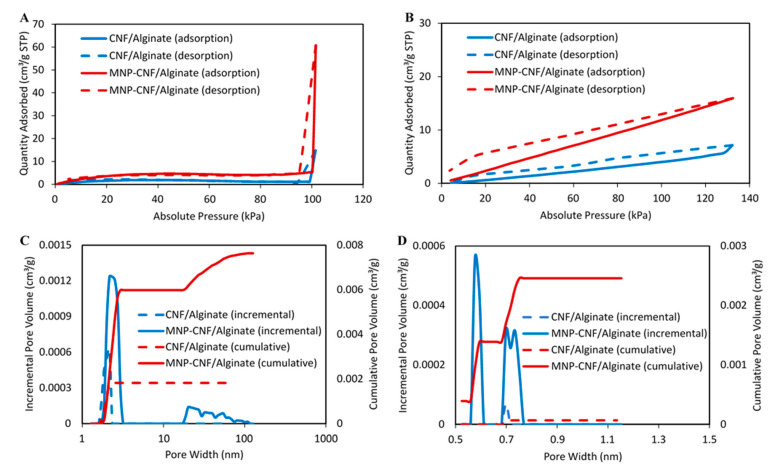
(**A**) N_2_ adsorption isotherm at −196 °C. (**B**) CO_2_ adsorption isotherm at 0 °C. (**C**) Incremental (main axis in blue) and cumulative pore volume (secondary axis in red) as a function of pore width calculated from N_2_ adsorption isotherm. (**D**) Incremental (main axis in blue) and cumulative micropore (secondary axis in red) volume as a function of pore width calculated from CO_2_ adsorption isotherm of CNF/alginate and MNP–CNF/alginate.

Figure 5 shows the N_2_ and CO_2_ adsorption isotherms and the pore size distribution for the CNF/alginate and MNP–CNF/alginate gel beads. The linearity of the N_2_ adsorption isotherms represents a non-porous structure, while the deviation from linearity entails the presence of micro- or mesoporous systems [54]. In Figure 5A, the Type II isotherm showed variation from the linearity in the high-pressure region, suggesting the presence of microporous alginate in the beads [55]. The higher amount of nitrogen adsorbed by the MNP–CNF/alginate gel bead compared to the CNF/alginate gel bead in the relative high-pressure region is likely due to the presence of Fe_3_O_4_ in the matrix. Fe_3_O_4_ increases the heterogeneity of the adsorbent, leading to higher porosity and, consequently, a better nitrogen absorbency [56]. The isotherms’ N_2_ adsorption curve indicates step condensation behaviour which also could be due to the presence of microporous structures in the beads [57].

Table 2 summarises the pore volume and surface area of the nanocomposite beads. It has been shown that the nitrogen volume adsorbed at equilibrium is regulated by the accessible micropore volume rather than by the internal surface area. Correspondingly, a higher uptake was observed for the MNP–CNF/alginate than the CNF/alginate gel beads, showing that the use of MNPs can lead to a significant increase in micropore volume, which is consistent with the results presented in Table 2. Figure 5C shows the pore size distribution of the gel beads in the range 1–150 nm. The CNF/alginate gel beads exhibited a less-developed pore structure. The pores were only observed with a size of around 2 nm, and the cumulative pore volume was 0.002 cm^3^/g. On the other hand, the MNP–CNF/alginate gel beads showed a relatively better-developed pore structure in terms of pore fraction, size distribution, pore volume, and connectivity. For the MNP–CNF/alginate gel beads, the cumulative pore volume of the pores with a size around 2 nm was 0.006 cm^3^/g, which was three times larger than that of the CNF/alginate gel beads. In addition, larger pores (30–100 nm) were also observed on the MNP–CNF/alginate gel beads, accounting for 21% of the total cumulative pore volume.

The CO_2_ adsorption capacity for the CNF/alginate and MNP–CNF/alginate gel beads is shown in Figure 5B. The curves go upward at low pressure (P < 120 kPa) and are almost linear, suggesting that the nanocomposite beads have similar pore characteristics during the adsorption and desorption of CO_2_. MNP–CNF/alginate adsorbed a higher amount of CO_2_ because of the larger surface area and porosity. The reflection of the adsorption and the filling of gas in micropores can be used to analyse the micropores [55,58]. The pore size distribution of the gel beads was found to be in the range 0.5–1.5 nm (Figure 5D). The CNF/alginate gel beads exhibited almost no porosity in this size range. On the other hand, the MNP–CNF/alginate gel beads showed pores with sizes from 0.6 to 0.8 nm. The cumulative pore volume of the MNP–CNF/alginate gel beads in this size range was 0.0025, which was 36 times larger than that of the MNP–CNF/alginate gel beads. Adding the magnetic nanoparticles into the gel beads not only increased the surface area but also introduced micropores, mesopores, and macropores into the matrix.

### 3.2. Pollutant Adsorption from Wastewater

The pollutant concentrations in the wastewater before and after adsorption were characterised using ICP-AES at ALS Environmental Division, Brisbane, Australia (Table 3). The concentrations of aluminium, potassium, selenium, sodium, vanadium, and sulphur show varying degrees of decline after the adsorption on the gel beads. The adsorption capacity of these pollutants on the beads was calculated as well. It is worth mentioning that due to the different initial concentrations of the various pollutants, the adsorption capacity of the pollutants should be evaluated as a reference for comparison between the different beads. The pollutant adsorption was possibly due to the integrated effects of coordination and electrostatic interactions during the removal of the pollutants using the gel beads [59]. Overall, the MNP–CNF/alginate gel beads show a higher adsorption capacity than the CNF–alginate ones. The MNP–CNF/alginate gel beads have more active adsorption centres for binding to pollutants, thereby increasing their adsorption capacity. In addition, the larger surface area and well-developed microporous structure of the MNP–CNF/alginate gel beads further increase its water treatment efficiency.

## 4. Conclusions

Hybrid nanocomposites of CNF/alginate and MNP–CNF/alginate hydrogel beads were prepared and studied by analysing their morphology, swelling behaviour, thermal and mechanical strength, and pore structure. The lowest water swelling achieved for the MNP–CNF/alginate beads is owing to the addition of magnetic nanoparticles onto the reinforcing cellulose nanofibres.

The composite beads have a high compressive stress resistance, particularly the MNP–CNF/alginate beads, providing > 3.5% stress tolerance at ~82% compressive strain when a usual alginate bead breaks. A fully reversible stress-strain curve was obtained upon loading and unloading for both CNF/alginate and MNP–CNF/alginate beads, indicating the reusability of the gel beads in a packed column-based continuous water treatment process. According to the N_2_ adsorption/desorption isotherm, the MNP–CNF/alginate gel beads adsorbed a maximum amount of N_2_ compared to the CNF/alginate gel beads in the relative high-pressure region. Both gel beads had pores smaller than 10 nm (MNP–CNF/alginate also possessed pores in the range 30–100 nm), and the CNF/alginate gel demonstrates a more homogeneous pore structure than MNP–CNF/alginate.

The adsorption capacity of both gel beads was found to be competent at a lower concentration of pollutants, including aluminium, potassium, selenium, sodium, vanadium, and sulphur. A higher adsorption capacity was observed for the MNP–CNF/alginate gel beads than the CNF/alginate beads due to their higher active centres for attachment of the pollutants. It is worth noting that the current work uses 50:50 volume ratio (as a basis) when mixing alginate with CNF or MNP–CNF solution. The high filler concentration resulted in a high diffusion barrier for water penetration into the nanocomposite beads. Lowering the filler concentration in the gel beads would give better adsorption results but at a compromised compressive strength. Compositional optimisation is hence needed, which we anticipate performing in our future research work.

Overall, composite gel beads emerged with promising features for their application in the wastewater treatment process due to their physical and mechanical properties as well as their adsorption of pollutants (aluminium, potassium, selenium, sodium, vanadium, sulphur). In contrast to conventional pollutant adsorbents that are toxic, the developed gel adsorbent is safe for users as well as environmentally friendly. While the strength of the current article lies in fundamental studies on pollutant adsorption and the robustness of nanocomposite beads, our future work will focus more on applied research, including packed column-based adsorption and regeneration studies. We also intend to utilise several analytical (e.g., FTIR and UV-Vis) and X-ray (e.g., XRD and XPD) techniques to evaluate pre- and post-adsorption and regeneration experiments. It is also essential to assess the antimicrobial properties of the gel beads to enhance user confidence in safety, and this is one of our future research goals.

## Figures and Tables

**Table 1:** Comparative compressive strength and application fields of MNP–CNF/alginate beads with some previously published research.

Composite Gel	Compressive Strength (kPa)	Potential Application	References
MNP–CNF/Alginate	>853	Wastewater treatment (pollutant adsorption)	This work
CNF/Collagen	~190	Biomedical (tissue engineering)	[47]
CNF/Polyethylene glycol	~500	Biomedical (cartilage repair)	[48]
CNF/Poly-N-isopropylacrylamide	~66	Biomedical	[49]
CNF/chitosan	~30	Biomedical (tissue engineering)	[50]
CNF/Polyacrylamide-Alginate	~290	Wastewater treatment (pollutant adsorption)	[51]

**Table 2:** Microporous properties of composite gel beads.

Sample	N_2_ Adsorption		CO_2_ Adsorption		
	Total Pore Volume (cm^3^/g)	BET Specific Surface Area (m^2^/g)	Equivalent Surface Area (m^2^/g)	Limiting Micropore Volume (cm^3^/g)	Limiting Micropore Capacity: (cm^3^/g STP)
CNF/Alginate	0.0018	7.01	408.47	0.2702	174.66
MNP–CNF/Alginate	0.0080	17.02	893.12	0.6081	393.16

**Table 3:** Pollutant concentration in wastewater before and after the adsorption by gel beads.

Compound	Normalised Wastewater Concentration (mg/L)			Adsorption Capacity of Gel Beads (mg/g)	
	Before Adsorption	After Adsorption		After Adsorption	
		CNF/Alginate	MNP–CNF/Alginate	CNF/Alginate	MNP–CNF/Alginate
Al	100	40	14	1.22	22
K	100	94.73	79.5	6.6	13.2
Se	100	88	75.7	14.3	19
Na	100	93.51	81.8	8.8	11.1
V	100	94	66	11.1	44.4
S	100	92	70.8	9.8	13.7

## Data Availability

The data that support the findings of this study are available within this article. Additional data are available from the corresponding authors on request.
